# Adjunctive Integrated Stress Response Inhibition Accelerates Tuberculosis Clearance in Mice

**DOI:** 10.1128/mbio.03496-22

**Published:** 2023-02-28

**Authors:** Stefanie Krug, Pankaj Prasad, Shiqi Xiao, Shichun Lun, Camilo A. Ruiz-Bedoya, Mariah Klunk, Alvaro A. Ordonez, Sanjay K. Jain, Geetha Srikrishna, Igor Kramnik, William R. Bishai

**Affiliations:** a Center for Tuberculosis Research, Johns Hopkins University School of Medicine, Baltimore, Maryland, USA; b Department of Medicine, Johns Hopkins University School of Medicine, Baltimore, Maryland, USA; c Center for Infection and Inflammation Imaging Research, Johns Hopkins University School of Medicine, Baltimore, Maryland, USA; d Department of Pediatrics, Johns Hopkins University School of Medicine, Baltimore, Maryland, USA; e The National Emerging Infectious Diseases Laboratory, Boston University School of Medicine, Boston, Massachusetts, USA; f Department of Medicine, Pulmonary Center, Boston University School of Medicine, Boston, Massachusetts, USA; Washington University in St. Louis

**Keywords:** *Mycobacterium tuberculosis*, tuberculosis drug therapy, ISRIB, host-directed therapy, inflammation, integrated stress response, tuberculosis drug

## Abstract

Despite numerous advances in tuberculosis (TB) drug development, long treatment durations have led to the emergence of multidrug resistance, which poses a major hurdle to global TB control. Shortening treatment time therefore remains a top priority. Host-directed therapies that promote bacterial clearance and/or lung health may improve the efficacy and treatment duration of tuberculosis antibiotics. We recently discovered that inhibition of the integrated stress response, which is abnormally activated in tuberculosis and associated with necrotic granuloma formation, reduced bacterial numbers and lung inflammation in mice. Here, we evaluated the impact of the integrated stress response (ISR) inhibitor ISRIB, administered as an adjunct to standard tuberculosis antibiotics, on bacterial clearance, relapse, and lung pathology in a mouse model of tuberculosis. Throughout the course of treatment, ISRIB robustly lowered bacterial burdens compared to the burdens with standard TB therapy alone and accelerated the time to sterility in mice, as demonstrated by significantly reduced relapse rates after 4 months of treatment. In addition, mice receiving adjunctive ISRIB tended to have reduced lung necrosis and inflammation. Together, our findings identify the ISR pathway as a promising therapeutic target with the potential to shorten TB treatment durations and improve lung health.

## INTRODUCTION

Despite the development of a highly effective first-line treatment regimen, tuberculosis (TB) remains one of the top 10 causes of death worldwide and a leading cause of death by an infectious agent. In 2020, approximately 10 million people developed TB, resulting in over 1.5 million human deaths (1). Contributing factors to the persistently high disease prevalence include multidrug resistance, HIV coinfections, and lengthy treatment durations (6 months), which hamper accessibility, implementation, and adherence. Shorter treatment durations could greatly improve global TB control but may require a better understanding of TB pathogenesis and the targeting of previously unexplored pathways.

TB is a chronic, often lifelong disease, caused by infection with Mycobacterium tuberculosis. Infection triggers elaborate pro- and anti-inflammatory responses that inadvertently promote not just disease containment but also long-term bacterial persistence within the host (2–5). These responses are attractive targets for host-directed therapies (HDTs) aiming to improve the elimination of the infection (6–9). A prime example is the hallmark TB granuloma, an immune microenvironment that contains the infection but also enables the prolonged survival and ultimate transmission of M. tuberculosis (10). The cytokine tumor necrosis factor (TNF) is considered crucial for granuloma formation and maintenance and essential for TB containment (11). However, in TB-susceptible genotypes, prolonged TNF stimulation promotes the necrosis of M. tuberculosis-infected cells through the generation of reactive oxygen species (12, 13), the proteotoxic stress response, and the superinduction of type I interferon (IFN) pathways, culminating in the aberrant activation of the integrated stress response (ISR) (14). The ISR is a protective host response to viral infections and other stressors, aimed at restoring cellular homeostasis by modulating global protein synthesis via the phosphorylation of eukaryotic translation initiation factor 2 alpha (eIF2α) (15); however, excessive or prolonged ISR activation can instead trigger cell death (16).

While its role in bacterial infections is just emerging (17), the ISR transcription factor ATF3 is highly abundant in murine and human TB granulomas, especially in cells surrounding the necrotic core (18). We recently discovered a causal relationship between type I IFN-driven aberrant ISR activation, TB susceptibility, and granuloma necrotization (14). Promisingly, ISR inhibition with the eIF2α phosphorylation inhibitor ISRIB reduced granuloma necrosis, lung inflammation, and bacterial numbers in M. tuberculosis-infected mice over the course of 8 weeks (14). ISRIB is in development for human use and has shown therapeutic benefits in preventing age-related memory loss (19) and in neurodegenerative syndromes, including Alzheimer’s disease, Parkinson’s disease, and amyotrophic lateral sclerosis (20–23). Here, we examined the therapeutic potential of ISR inhibition in the context of standard TB chemotherapy. We demonstrated that adjunctive ISRIB significantly reduced bacterial burdens and relapse rates in mice while exerting protective effects on lung health. Therefore, ISRIB may offer therapeutic benefits and shorten the duration of TB treatment.

## RESULTS

### Adjunctive ISRIB administration reduces lung bacterial burden in M. tuberculosis-infected mice.

**FIG 1:**
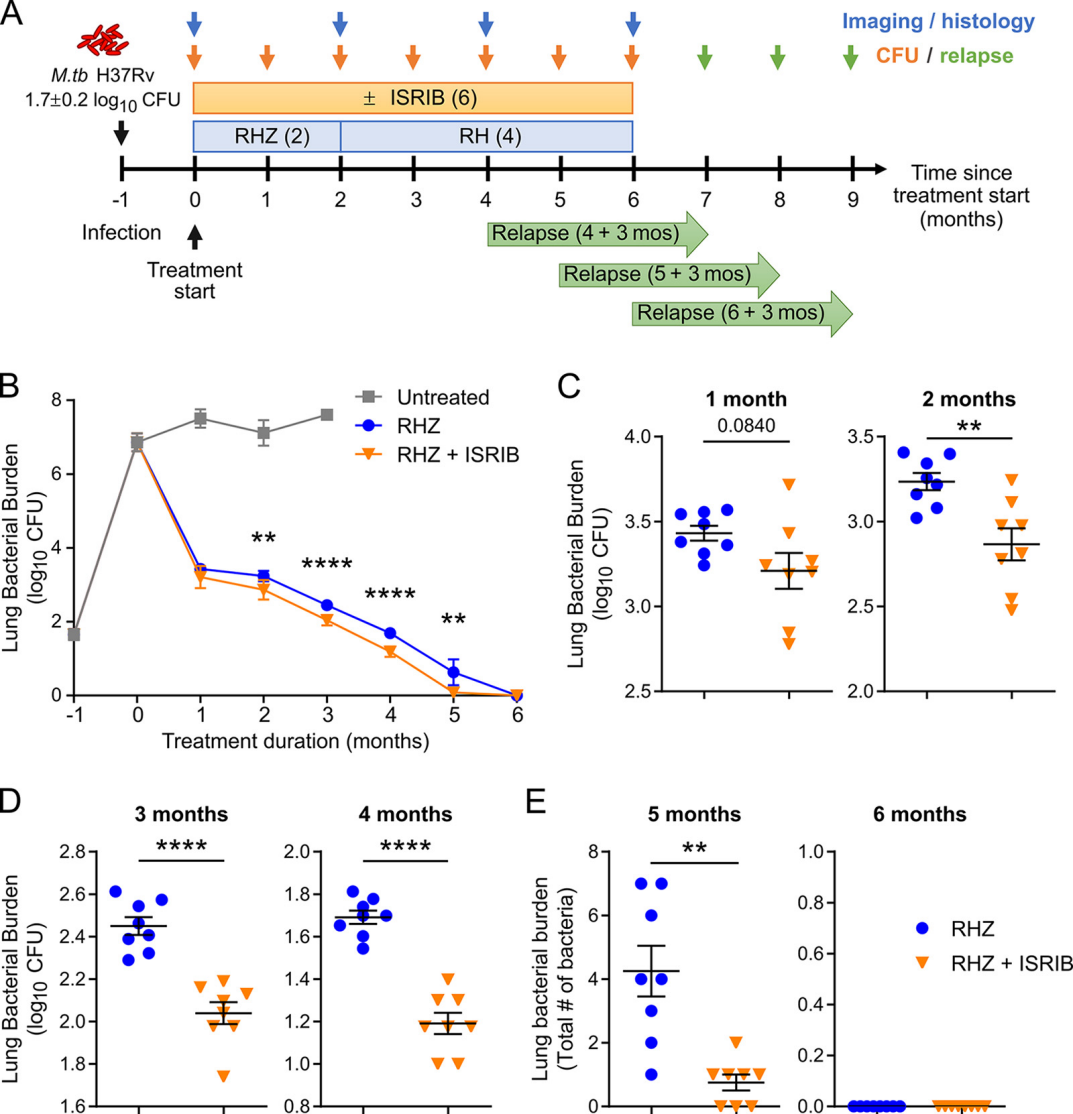
Adjunctive inhibition of the integrated stress response (ISR) potentiates standard TB therapy. (A) Study overview. Female C3HeB/FeJ mice were aerosol infected with M. tuberculosis H37Rv (implantation of 1.65 ± 0.15 log_10_ CFU). Starting 28 days postinfection, mice received standard TB antibiotics (RHZ; 10 mg/kg rifampin [R], 10 mg/kg isoniazid [H], and 150 mg/kg pyrazinamide [Z] for 2 months, followed by 4 months of RH) with or without the integrative stress response inhibitor ISRIB (0.25 mg/kg) 5 days/week for up to 6 months. Bacterial burdens were evaluated at 1-month intervals (*n* = 8). After 4, 5, and 6 months of treatment, 15 mice from each group were held without treatment for an additional 3 months to determine relapse rates (green arrows). Lung inflammation and pathology were evaluated at the start of and after 2, 4, and 6 months of treatment by ^18^F-FDG PET/CT and histology, respectively. (B) Time course of lung bacterial burdens (mean log_10_ CFU ± SEM) in untreated, RHZ-treated, or RHZ+ISRIB-treated mice. (C to E) Lung bacterial burdens in RHZ- or RHZ+ISRIB-treated mice after 1 to 6 months of treatment. Each symbol represents the log_10_ CFU (C and D) or absolute CFU (E) in an individual mouse. Horizontal lines indicate group mean values ± SEM. Statistical differences in log_10_-transformed (1 to 4 months) or absolute (5 months) CFU in RHZ- and RHZ+ISRIB-treated mice were evaluated by unpaired two-tailed *t* test (2, 3, and 4 months), with Welch’s correction for unequal variance where indicated (1 and 5 months). **, *P < *0.01; ***, *P < *0.001; ****, *P < *0.0001. The addition of ISRIB potently reduced bacterial burdens compared to RHZ alone throughout the course of treatment.

ISRIB, a highly potent ISR inhibitor with an intracellular 50% effective concentration (EC_50_) of 5 nM, exhibits good pharmacokinetic properties and bioavailability (19, 24). It resets proteostasis induced by chronic inflammation in macrophages (14, 25). In M. tuberculosis-infected B6.Sst1^S^ mice, which are known to form necrotic TB granulomas, we found that ISRIB was effective as a monotherapy and reduced bacterial numbers, lung inflammation, and granuloma necrosis even though ISRIB had no direct bactericidal activity against M. tuberculosis (14). Here, we set out to determine if this therapeutic benefit was preserved when ISRIB (0.25 mg/kg of body weight) was given in combination with a standard course of the first-line TB antibiotics, rifampin (R; 10 mg/kg for 6 months), isoniazid (H; 10 mg/kg for 6 months), and pyrazinamide (Z; 150 mg/kg for 2 months) (RHZ), in a mouse model of chronic TB with a different genetic background (Fig. 1A). We carried out the study in C3HeB/FeJ mice, since these mice have been extensively validated as a preclinical mouse model of TB and develop heterogenous cellular and necrotic granulomatous lung lesions following M. tuberculosis infection that resemble those observed in human TB patients, nonhuman primates, rabbits, and guinea pigs (26). While the *sst1^S^* allele, which controls granuloma necrotization, is present in both B6.Sst1^S^ and C3HeB/FeJ mice, the latter exhibit even greater TB susceptibility, bacterial burdens, and immunopathology due to the presence of additional minor susceptibility loci (27–29). Since C3HeB/FeJ mice recapitulate the range of disease pathology associated with human TB, the effects of adjunctive ISRIB in this model may be more reflective of the potential effects in humans. In the chronic model, infection is allowed to establish for 4 to 6 weeks prior to the initiation of antibiotic or host-directed therapy, which more closely resembles the clinical situation and allows us to probe the therapeutic impact of ISRIB on established granulomas. The efficacy of combination therapy with and without ISRIB was determined by evaluating bacterial clearance, time to sterility, lung pathology, and inflammation. To assess the treatment-shortening potential of adjunctive ISRIB therapy, separate groups of mice were treated for 4, 5, or 6 months and held off treatment for an additional 3 months following drug administration before evaluating relapse rates.

At the start of treatment, the mean lung bacterial burden was 6.9 ± 0.2 log_10_ CFU (mean ± standard error of the mean) (Fig. 1B). Infected untreated mice were moribund 4 months after M. tuberculosis challenge and were euthanized. After the first month of treatment, the mean lung CFU declined to 3.4 ± 0.1 log_10_ CFU in mice receiving RHZ, and the CFU counts continued to decline until all mice were culture negative after 6 months of treatment (Fig. 1C to E). Even in the context of this highly bactericidal RHZ regimen, the addition of ISRIB (RHZ+ISRIB) robustly enhanced bacterial clearance throughout and significantly lowered lung CFU counts compared to RHZ alone, from 3.2 ± 0.3 log_10_ CFU after the first month of treatment with RHZ+ISRIB (levels seen only after 2 months of treatment with RHZ alone) to 2.87± 0.27 log_10_ CFU after 2 months of treatment with RHZ+ISRIB treatment. Interestingly, after 5 months of treatment, three mice receiving RHZ+ISRIB had no detectable bacilli in their lungs, while all RHZ-treated mice had quantifiable bacterial burdens, suggesting that the addition of ISRIB to the first-line regimen may accelerate time to sterility (Fig. 1E).

### Addition of ISRIB potentiates standard TB therapy and shortens duration of TB treatment.

**FIG 2:**
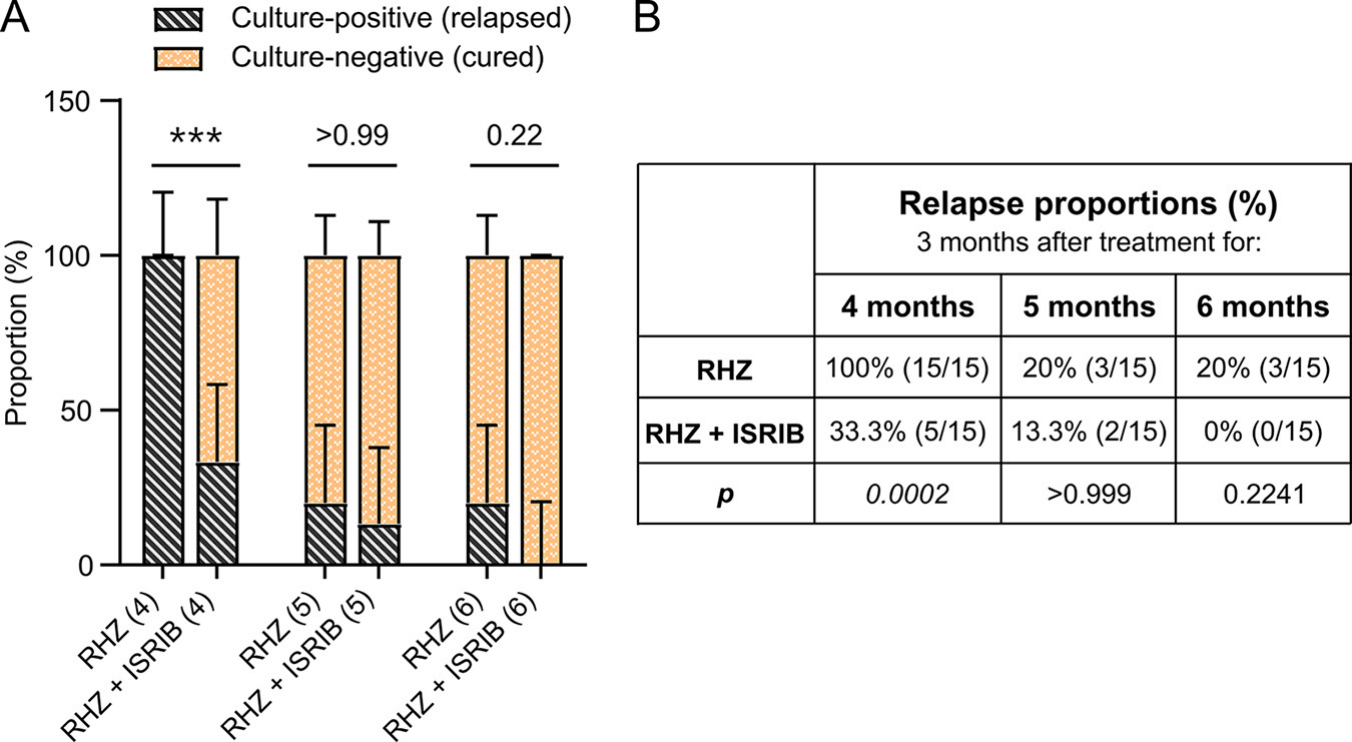
Adjunctive inhibition of the integrated stress response shortens TB treatment duration. Relapse rates in C3HeB/FeJ mice treated with RHZ with or without the integrative stress response inhibitor ISRIB (*n* = 15). Mice were treated for 4, 5, or 6 months and held for an additional 3 months without treatments before they were sacrificed and the entire lung homogenized and plated. Mice were considered cured if no colonies were detected after incubating plates for 8 weeks and relapsed if any colonies were found. (A) Proportions of cured (culture negative) and relapsed (culture positive) mice after 2, 4, and 6 months of treatment. Data are presented in stacked columns as fractions of the total (%), with error bars indicating 95% confidence intervals computed by the hybrid Wilson-Brown method. The proportions of relapsed mice were compared by two-sided Fisher’s exact test. (B) Table showing relapse percentages, the number of culture-positive mice over the total number of mice for each group, and the *P* value calculated for each time point. After 4 months, mice receiving adjunctive ISRIB had significantly lower relapse rates than mice receiving only RHZ. ***, *P < *0.001.

To evaluate the sterilizing effect of adjunctive ISRIB treatment, we also calculated the proportion of mice with culture-positive relapse 3 months after the completion of 4, 5, or 6 months of the indicated drug regimen (Fig. 1A). Mice were considered cured if no colonies were found 8 weeks after plating and relapsed if any colonies were found. For mice that received 4 months of the standard regimen (RHZ), there was 100% relapse (15/15), with culture-positive lungs 3 months after the completion of treatment, while relapse was significantly reduced, to 33.3% (5/15), in mice that received RHZ+ISRIB (Fig. 2). After 6 months of treatment, relapse-free cure was observed in all mice that received RHZ+ISRIB, while 20% of mice (3/15) that received only the standard regimen still relapsed, indicating that the addition of ISRIB could shorten treatment compared to the control regimen and, more importantly, lead to complete sterilization 6 months after treatment.

### Adjunctive inhibition of ISR reduces inflammation and necrosis in mouse lungs.

**FIG 3:**
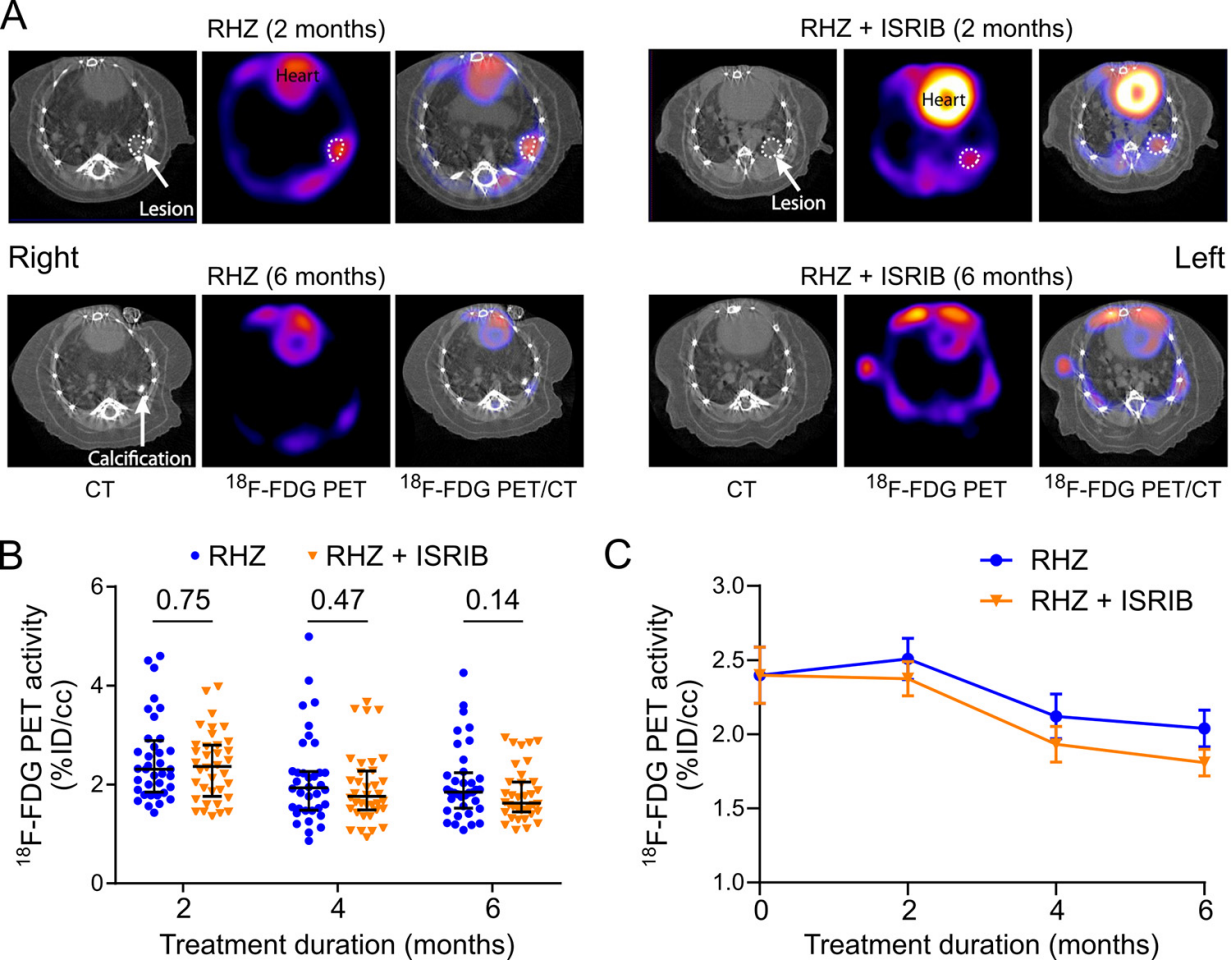
Adjunctive inhibition of the integrated stress response modestly lowers lung inflammation. Lung inflammation in RHZ- and RHZ+ISRIB-treated C3HeB-FeJ mice evaluated by ^18^F-FDG PET/CT (*n* = 6). (A) Paired transverse sections (CT, ^18^F-FDG PET, and merged ^18^F-FDG PET/CT) in a representative RHZ-treated (left) or RHZ+ISRIB-treated (right) mouse after 2 (top) and 6 (bottom) months of treatment. TB lesions are highlighted with dotted lines. One mouse from the RHZ group persisted with a calcified lesion. (B) Scatterplots of ^18^F-FDG activity per region of interest (ROI) after 2, 4, and 6 months of treatment (6 mice/group, 6 ROIs/mouse). PET data are presented on a linear scale with median values ± interquartile ranges indicated. Differences were assessed by uncorrected Mann-Whitney test. (C) ^18^F-FDG activities (mean values ± SEM) calculated from 6 ROIs per animal. While no differences were noted in the ^18^F-FDG activities at these four time points, RHZ+ISRIB-treated mice consistently had lower mean levels of ^18^F-FDG activity than mice treated with RHZ only.

^18^F-fluorodeoxyglucose (^18^F-FDG) accumulates in neutrophils and macrophages at sites of inflammation (30, 31). ^18^F-FDG positron emission tomography coregistered with computed tomography (PET/CT) is therefore extensively used as an imaging modality for evaluating TB lesions in animal models and in patients and to monitor the bactericidal activity of drugs in preclinical studies (32–35). We therefore evaluated lung inflammation in untreated, RHZ-treated, and RHZ+ISRIB-treated mice by serial ^18^F-FDG PET/CT imaging (32). Well-defined foci of ^18^F-FDG uptake colocalizing with TB lesions were observed in lung fields of infected mice, as shown in representative mouse lungs after 2 and 6 months of treatment (Fig. 3A). Although the levels of ^18^F-FDG activity calculated from 6 regions of interest (ROIs) per animal did not differ significantly between RHZ- and RHZ+ISRIB-treated mice after 2, 4, or 6 months of treatment, the latter group exhibited consistently lower mean values for ^18^F-FDG activity than RHZ-only treated mice, suggesting that the addition of ISRIB further reduced inflammation in RHZ-treated mice (Fig. 3B and C).

**FIG 4:**
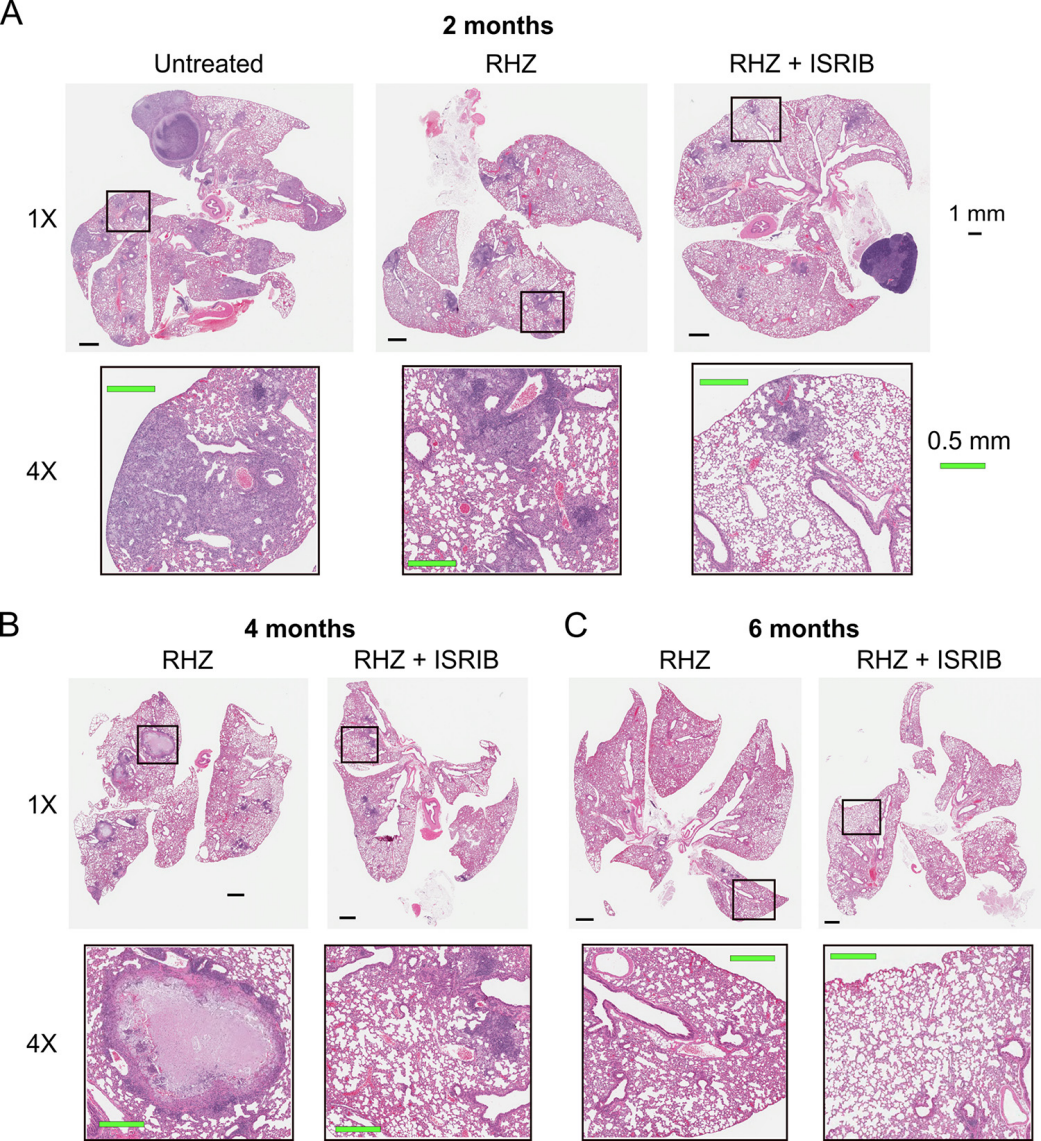
Adjunctive inhibition of the integrative stress response may reduce necrosis in mouse lungs. (A to C) Representative H&E-stained lung sections of M. tuberculosis-infected C3HeB/FeJ mice treated with RHZ or RHZ+ISRIB for 2 (A), 4 (B), or 6 (C) months or of untreated mice 3 months postinfection (control for 2 months of treatment) (A). The top row of each panel shows low-magnification (1×) images of entire lung sections. The bottom row of each panel shows higher-magnification (4×) images of features outlined in the corresponding low-magnification image. Necrotic granulomas were observed in untreated and RHZ-treated mice but not in RHZ+ISRIB-treated mice.

Since the lower levels of ^18^F-FDG activity in the lungs of RHZ+ISRIB-treated mice indicated reduced inflammation and ISRIB monotherapy led to a significant decrease in necrotic granuloma formation and overall lung destruction 8 weeks postinfection, as we reported earlier (14), we performed histological evaluation of inflammation and necrosis in untreated and treated mice. As seen in representative hematoxylin and eosin (H&E)-stained lung sections (Fig. 4; Fig. S1 and S2 in the supplemental material) of M. tuberculosis-infected mice treated with RHZ ± ISRIB for 2, 4, or 6 months or of untreated mice 3 months postinfection (control for 2 months treatment), necrotic granulomas were present in the lungs of untreated and RHZ-treated mice but absent in any mice receiving adjunctive ISRIB treatment. These findings suggest that the addition of ISRIB to conventional TB therapy may reduce lung inflammation, thereby restoring normal lung structure and function.

10.1128/mbio.03496-22.1 FIG S1 H&E-stained lung sections. Low-magnification (1×) images of H&E-stained lung sections of M. tuberculosis-infected C3HeB/FeJ mice treated with RHZ or RHZ+ISRIB for 2, 4, or 6 months or of untreated mice 3 months postinfection (control for 2 months of treatment). Scale bar indicates 1 mm. Necrotic granulomas were observed in untreated and RHZ-treated mice but not in RHZ+ISRIB-treated mice. Download FIG S1, PDF file, 0.3 MB. Copyright © 2023 Krug et al. 2023 Krug et al. https://creativecommons.org/licenses/by/4.0/ This content is distributed under the terms of the Creative Commons Attribution 4.0 International license.

10.1128/mbio.03496-22.2 FIG S2 H&E-stained lung sections. High-magnification (4×) images of H&E-stained lung sections of M. tuberculosis-infected C3HeB/FeJ mice treated with RHZ or RHZ+ISRIB for 2, 4, or 6 months or of untreated mice 3 months postinfection (control for 2 months of treatment). Scale bar indicates 0.5 mm. Necrotic granulomas were observed in untreated and RHZ-treated mice but not in RHZ+ISRIB-treated mice. The addition of ISRIB may preserve functional airspace in mice. Download FIG S2, PDF file, 0.4 MB. Copyright © 2023 Krug et al. 2023 Krug et al. https://creativecommons.org/licenses/by/4.0/ This content is distributed under the terms of the Creative Commons Attribution 4.0 International license.

## DISCUSSION

Granulomas are the pathological hallmark of TB. Necrotic granulomas, arising from the necrosis of infected macrophages, contribute significantly to disease progression and pathogen spread (36–38). Host cell necrosis is a mechanism exploited by M. tuberculosis that promotes bacterial replication, dissemination, and immune evasion (39–41). In fact, M. tuberculosis has been shown to replicate inside (pre)necrotic macrophages (42), and preventing necrosis is associated with enhanced M. tuberculosis clearance in macrophages and in mice (14). Several recent studies have greatly advanced our understanding of the necrotic mechanism underlying TB susceptibility. The progression from latent infection to active TB disease is the result of an imbalanced rather than a deficient immune response, characterized by a tumor necrosis factor-alpha (TNF-α)-dependent increase in type I IFN production that drives ISR hyperactivation and the necrotization of lung lesions (14, 43, 44). In the majority of infected humans and in resistant mouse strains, such as C57BL/6 mice, this process is held in check by the nuclear protein SP110/Ipr1, which is encoded within the *sst1* locus and prevents ISR induction by downregulating NF-κB and TNF-α signaling (14, 44). Any defect in this regulation is strongly associated with granuloma necrotization and TB severity in both humans and mice, as exemplified by C3HeB/FeJ mice, which carry the *sst1^S^* allele that does not express SP110/Ipr1 (27, 28, 44, 45). ISR markers are abundantly expressed in necrotic but not nonnecrotic human TB granulomas, underscoring the importance of ISR activation in TB progression and transmission (18). We therefore reasoned that pharmacological ISR inhibition may overcome this regulatory deficit and restore TB resistance in susceptible individuals.

Indeed, our prior monotherapy studies revealed that ISRIB treatment reduced bacterial loads, inflammation, and necrosis in M. tuberculosis-infected mouse lungs compared to the lungs of untreated mice (14). Since ISRIB had no detectable direct bactericidal effect on M. tuberculosis proliferation in culture (MIC of >256 μg/mL) or in macrophages (14), these protective effects of ISRIB monotherapy were the sole product of its host-directed activity (i.e., inhibition of eIF2α phosphorylation). This validated ISR inhibition as a promising host-directed TB therapy and suggested that the addition of ISRIB to the TB regimen may shorten treatment time.

In this study, we therefore tested the efficacy of ISRIB as an adjunctive therapeutic in combination with the standard TB treatment regimen (RHZ) in C3HeB/FeJ mice, which lack the SP110/Ipr1 regulator and develop human-like necrotic lung lesions following M. tuberculosis infection and are therefore particularly appropriate for evaluating the efficacy of host-directed drugs aimed at de-escalating TB-induced aberrant stress responses. We found that ISRIB significantly increased bacterial clearance by the first-line anti-TB drugs when tested in combination, starting from 2 months of treatment, suggesting that ISRIB may accelerate time to sterility. The fact that adjunctive ISRIB accelerated bacterial clearance and reduced relapse even in the context of the highly effective standard TB therapy suggests that ISR activation continues throughout treatment and may even antagonize the efficacy of TB antibiotics, potentially by promoting bacterial replication and dissemination through expanding necrosis. This beneficial effect of ISRIB was accompanied by reductions in inflammation and necrosis in infected lungs compared with the lungs of mice treated with TB antibiotics alone. While there were highly significant differences in bacterial burdens between 2 and 5 months of treatment with RHZ versus RHZ+ISRIB, sterility was achieved in both groups only at 6 months. This could suggest that higher doses of the inhibitor might provide significant treatment shortening. However, we had found earlier that ISRIB at 0.25 mg/kg (the dose used in the present study) demonstrated superior inhibition of M. tuberculosis proliferation in B6.Sst1S mice compared to 1.0 mg/kg. Higher doses (5 mg/kg) were in fact deleterious (14). While we have not formally proven that the action is specific to mice that develop necrotic lesions (for example, by testing ISRIB in wild-type mice, such as C3H or C57BL/6), our prior results suggest that necrosis development is required for ISRIB’s activity (14). We only saw a moderate decrease in PET activity over the 6-month course of standard TB therapy compared to the untreated baseline, indicating that the dynamic range of detection in our study may have been insufficiently sensitive to capture the differences in inflammation between our two treatment arms. Despite this limitation, the mean PET activity was lower in RHZ+ISRIB treatment than in RHZ treatment alone at every time point, and we speculate that the differences in metabolic activity between RHZ and adjunctive ISRIB treatment may be more pronounced in a system with a greater dynamic PET/CT range (e.g., humans or rabbits). Together, these findings suggest that adjunctive ISRIB has the potential to benefit a substantial proportion of TB patients, but additional details, such as optimal ISRIB timing and dosing strategies, will need to be worked out prior to evaluating the safety and efficacy of ISRIB adjunctive therapy in human TB patients. Our results are also applicable to infected nonhuman primates, which exhibit granulomatous and necrotic lung pathology resembling human TB disease, and hence, our results in C3HeB/FeJ mice portend efficacy of ISRIB as an adjunctive therapeutic in primate studies and human patients in accelerating bacterial clearance and reducing treatment time.

Importantly, 6 months of ISRIB administration had no observable deleterious effects on mouse health or the efficacy of TB antibiotics, indicating that we were indeed able to “correct” TB susceptibility without compromising TB containment. This is in stark contrast to the other drivers of granuloma necrotization (IFNs, NF-κB, and TNF-α), which play critical roles in containing M. tuberculosis infection independently of necrosis formation and are thus poor candidates for host-directed therapies. That the ISR contributes primarily to TB progression and less to overall bacterial control is supported by our previous finding that in the early phase, the ISR was moderately activated in cells from both resistant and susceptible (*sst1^S^*) backgrounds, while the late-phase ISR hyperinduction was unique to the *sst1^S^* background (14). Since resistant mice successfully contain TB, ISR inhibition may not pose the same risks as the targeting of fundamental TB immune responses. However, since we did not evaluate cytokine or immune cell profiles in ISRIB-treated mice, we cannot rule out that ISRIB administration affected these immune responses as well.

The activation of the integrated stress response contributes to a wide range of pathologies, including neurodegeneration, cancers, and infections. Our studies suggest that ISRIB and other inhibitors that are being developed to mitigate ISR in these pathologies may also provide beneficial effects in the form of reduced granuloma necrosis and accelerated bacterial clearance, thereby reducing disease progression and spread in TB. Manipulation of ISR has also been reported for other bacterial infections (17). However, the activating pathway and resulting effects appear to be highly specific to the infecting organism. For example, Pseudomonas aeruginosa activated the ISR to reduce inflammatory responses and increase host cell viability, which may have contributed to bacterial survival in the host (46). In contrast, ISR activation by Shigella, Salmonella, and Listeria increased inflammatory responses but had divergent effects on the outcome of infection: ISR activation was host protective against Shigella and minimally protective against Salmonella but detrimental against Listeria by promoting bacterial replication (15). Similarly, ISR activation was required for the inflammatory response to Yersinia pseudotuberculosis but was directly manipulated by the bacteria as a means of immune evasion, and cells with defective eIF2a signaling were more susceptible to invasion by pathogenic Yersinia, Listeria monocytogenes, and Chlamydia trachomatis (47). In the context of TB, ISR activation is intertwined with the proinflammatory response to infection and drives a phenotype that benefits the pathogen rather than the host. It seems plausible that the ISR may be exploited as a virulence mechanism by pathogenic strains of M. tuberculosis to evade adaptive immune priming and ensure its dissemination and ultimate transmission. Dissecting this question may identify new druggable targets that could further improve the treatment of TB.

The global burden of TB remains unabated and substantial. While advances have been made in shortening the 6-month standard therapy (48) and in developing new regimens expected to be active against drug-resistant TB (49), there remain significant challenges in the chemotherapeutic management of TB. Here, we applied a mechanism-based precision medicine approach to develop a novel host-directed and antibiotic combination therapy with better efficacy than standard TB chemotherapy. Importantly, we have demonstrated that ISRIB can prevent granuloma necrotization and accelerate bacterial clearance in two different genetic backgrounds (B6.Sst1^S^ and C3HeB/FeJ) (14), both of which recapitulate the fundamental hallmark of human pulmonary TB. Targeting the host responses to the pathogen that allow its survival within granulomas may also circumvent antibiotic resistance, which would be a major advance toward overcoming TB treatment challenges. Together, our findings warrant further preclinical development of adjunctive ISRIB therapy for the treatment of TB in nonhuman primates.

## MATERIALS AND METHODS

Additional detail on the methods used is provided in Text S1.

10.1128/mbio.03496-22.3 TEXT S1 Additional details of the methods used in this work. Download Text S1, PDF file, 0.1 MB. Copyright © 2023 Krug et al. 2023 Krug et al. https://creativecommons.org/licenses/by/4.0/ This content is distributed under the terms of the Creative Commons Attribution 4.0 International license.

### Mouse infection.

All animal procedures were approved by the Institutional Animal Care and Use Committee of the Johns Hopkins University School of Medicine and carried out under biosafety level 3 (BSL3) containment. M. tuberculosis H37Rv was cultured in Middlebrook 7H9 broth (Gibco, Gaithersburg, MD) supplemented with 10% (vol/vol) oleic acid-albumin-dextrose-catalase (OADC; Difco, Thermo Fisher Scientific, Waltham, MA), 0.5% (vol/vol) glycerol, and 0.05% (vol/vol) Tween 80 (Sigma-Aldrich, St. Louis, MO). C3HeB/FeJ mice (Jackson Laboratory, Bar Harbor, ME) were aerosol infected with M. tuberculosis H37Rv using the Glas-Col inhalation exposure system (Glas-Col, Terre Haute, IN). Lung implantation was determined the day after infection (*n* = 3/run). Full details are in Text S1.

### Drug preparation and study arms.

Rifampin (R; 10 mg/kg), isoniazid (H; 10 mg/kg), and pyrazinamide (Z; 150 mg/kg) were purchased from Sigma-Aldrich and prepared weekly in distilled water. ISRIB (0.25 mg/kg) was dissolved in 45% saline, 50% polyethylene glycol 400 (PEG 400), and 5% dimethyl sulfoxide (DMSO) (50). All drugs were administered once daily, 5 days/week, by orogastric gavage (RHZ) and intraperitoneal injection (ISRIB).

One month postinfection, mice were randomly assigned to three treatment arms (outlined in Fig. 1A): standard TB therapy (RHZ; 2 months of RHZ treatment followed by 4 months of RH treatment), RHZ with adjunctive ISRIB (RHZ+ISRIB), and untreated (UNT). Lung bacterial burdens were quantified at 1-month intervals (RHZ and RHZ+ISRIB, *n* = 8; UNT, *n* = 5). For relapse studies, treatment was stopped after 4, 5, or 6 months and mice sacrificed 3 months later (*n* = 15). Full details are in Text S1.

### Bacterial enumeration and relapse determination.

Mice were sacrificed at predetermined intervals (see diagram in Fig. 1A). The lungs were aseptically removed, homogenized and serially diluted in phosphate-buffered saline (PBS), and spread on selective 7H11 agar (Difco, Thermo Fisher Scientific) plates. After 3 to 4 weeks, colonies were counted to determine total or log-transformed CFU per mouse. For relapse studies, the entire lung homogenate was plated undiluted. The presence of colonies on any plate (culture positive) indicated relapse, while no detectable growth on any plate after 8 weeks of incubation (culture negative) was considered cured. Full details are in Text S1.

### Histopathology.

Lungs were fixed in 10% neutral buffered formalin, paraffin embedded, sectioned, and H&E stained. Slides were digitally scanned (Aperio AT turbo scanner console version 102.0.7.5; Leica Biosystems, Vista, CA), transferred (Concentriq for Research version 2.2.4; Proscia, Philadelphia, PA), and visualized (Aperio ImageScope version 12.4.0.5043; Leica Biosystems Pathology Imaging, Buffalo Grove, IL). Histology images were assembled using the open-source vector graphics editor Inkscape for Windows version 0.92.4 (Boston, MA).

### ^18^F-FDG PET/CT imaging.

Six RHZ- or RHZ+ISRIB-treated mice were sequentially imaged after 0, 2, 4, and 6 months of treatment (32, 33, 51). Each animal was fasted, intravenously injected with 6.9 ± 0.69 MBq of ^18^F-FDG, and subjected to a 15-minute PET acquisition and subsequent CT scan (nanoScan PET/CT; Mediso, Arlington, VA). Regions of interest were manually selected using CT as a guide and applied to the PET data set using VivoQuant 2020 (Invicro, Boston, MA). Mean lung ^18^F-FDG PET activity per mouse was calculated as the average activity normalized by injected dose (% ID/mL). Full details are in Text S1.

### Statistical analysis.

The statistical tests used are indicated in the figure legends and were performed using Prism version 9.2.0 for Windows (GraphPad, San Diego, CA). Data represent mean values ± standard errors of the means (SEM) unless otherwise indicated. A *P* value below 0.05 was considered significant. Full details are in Text S1.
