# Supplementary material for: Adjunctive Integrated Stress Response Inhibition Accelerates Tuberculosis Clearance in Mice
Source: mBio. 2023 Feb 28;14(2):e03496-22. doi: 10.1128/mbio.03496-22 (PMC10128048; doi:10.1128/mbio.03496-22)
Supplement: FIG S1 [file mbio.03496-22-s0001.pdf]

Supplemental Figure S1

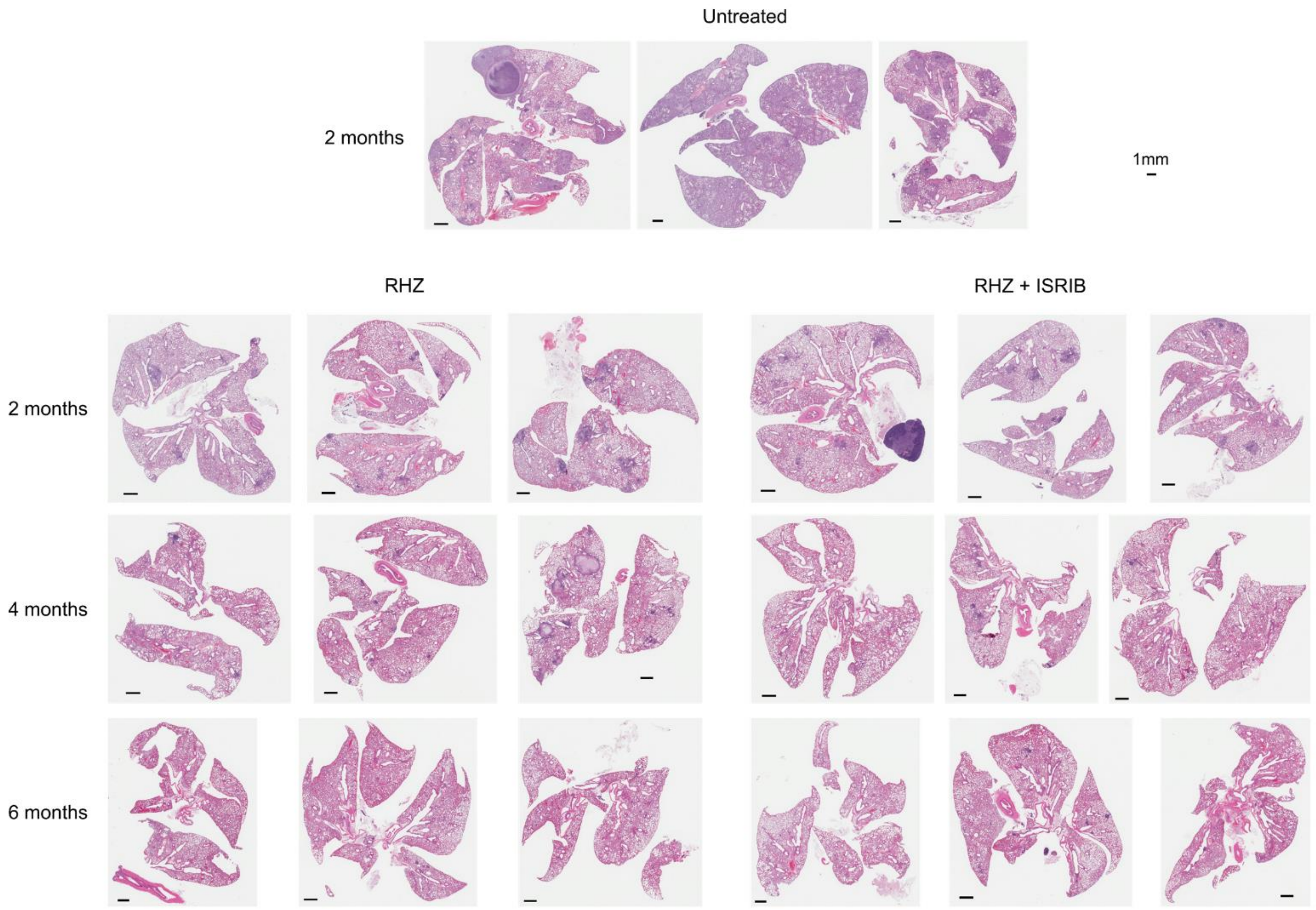

Supplemental Figure S1. H&E-stained lung sections (low magnification)

Low magnification (1X) images of H&E-stained lung sections of *Mtb*-infected C3HeB/FeJ mice treated with RHZ ± ISRIB for 2, 4, or 6 months, or untreated mice 3 months post-infection (control for 2 months treatment). Scale bar indicates 1 mm. Necrotic granulomas were observed in untreated and RHZ-treated, but not in RHZ + ISRIB-treated mice.
