# Supplementary material for: Adjunctive Integrated Stress Response Inhibition Accelerates Tuberculosis Clearance in Mice
Source: mBio. 2023 Feb 28;14(2):e03496-22. doi: 10.1128/mbio.03496-22 (PMC10128048; doi:10.1128/mbio.03496-22)
Supplement: TEXT S1 [file mbio.03496-22-s0003.pdf]

Adjunctive integrated stress response inhibition accelerates tuberculosis clearance in mice

Stefanie Krug[1,2,#], Pankaj Prasad[1,2,#], Shiqi Xiao[1,2], Shichun Lun[1,2], Camilo A. Ruiz-Bedoya[1,3,4], Mariah Klunk[1,3], Alvaro A. Ordonez[1,3,4], Sanjay K. Jain[1,3,4], Geetha Srikrishna[1,2], Igor Kramnik[5,6], William R. Bishai[1,2]*

ONLINE METHODS SUPPLEMENT

Mice

Six-week-old female C3HeB/FeJ (stock #658) mice were purchased from The Jackson Laboratory (Bar Harbor, ME, USA). Mice were maintained under ABSL3 conditions on a 12h:12h light:dark cycle, with rodent chow and water available *ad libitum*. All animal procedures were approved by the Institutional Animal Care and Use Committee of the Johns Hopkins University School of Medicine.

Bacteria

M. tuberculosis strain H37Rv was obtained from the Johns Hopkins Center for Tuberculosis Research, grown to an optical density at 600 nm of approximately 1.0 in Middlebrook 7H9 broth (Gibco Laboratories, Gaithersburg, MD, USA) supplemented with 10% (v/v) oleic acid-albumin-dextrose-catalase (OADC; BD Difco, Thermo Fisher Scientific, Waltham, MA, USA), 0.5% (v/v) glycerol and 0.05% (v/v) Tween 80 (Sigma-Aldrich, St. Louis, MO, USA) and stored in 1 ml aliquots at -80°C.

Aerosol infection

Mice were infected via the aerosol route using the Glas-Col Inhalation Exposure System (Terre Haute, IN). A fresh vial of *M. tuberculosis* was used for each infection, thawed on the day of infection and diluted in sterile phosphate-buffered saline (PBS, pH 7.4) at empirically determined factors to achieve the desired inoculum. On the day after infection, 3 mice per cycle were sacrificed to determine the number of CFUs implanted into the lungs. The general appearance and body weight of mice were monitored at least weekly throughout all experiments. All infections, housing of infected mice and handling of infectious materials were carried out under biosafety level 3 containment in dedicated facilities.

Drug preparation

Rifampin, isoniazid and pyrazinamide were purchased from Sigma-Aldrich (St. Louis, MO, USA). Solutions of rifampin (R; 10 mg/kg), and isoniazid (H; 10 mg/kg) and pyrazinamide (Z; 150 mg/kg) were prepared weekly in distilled water and stored at 4°C. The small molecule inhibitor of eIF2α phosphorylation, ISRIB (0.25 mg/kg) was dissolved in 45% saline, 50% PEG 400 and 5% DMSO (34). All drugs were administered once daily, five days/week, in a total volume of 0.2 ml per treatment. HZ and R were administered by orogastric gavage and ISRIB by intraperitoneal (*i.p.*) injection. The HZ solution was gently heated in a 55°C water bath and vortexed to dissolve prior to treating mice, and administered at least 1 h after R to reduce drug interactions.

Treatment arms and study design

One month after infection, mice were randomly assigned to three treatment arms: standard TB therapy ("RHZ"; 2 months RHZ followed by 4 months RH), standard TB therapy plus adjunctive integrated stress response inhibition ("RHZ + ISRIB"; 2 months RHZ + ISRIB followed by 4 months RH + ISRIB), and untreated (UNT). A subset of mice from each group (UNT: n=5; RHZ ± ISRIB: n=8) were sacrificed at one-month intervals for bacterial enumeration for the first 3 months of treatment, after which point most untreated mice appeared moribund and were euthanized. To evaluate potential treatment-shortening effects of adjunctive ISR inhibition, relapse rates were determined after 4, 5 and 6 months of treatment with RHZ or RHZ + ISRIB (n=15). For relapse studies, mice were sacrificed 3 months after treatment was stopped and the entire lung homogenate from each mouse was plated undiluted on selective 7H11 agar plates (as described below). The presence of colonies on any plate ("culture-positive") indicated

relapse while no detectable growth on any plate after 8 weeks of incubation ("culture-negative") was considered cured.

Tissue collection and bacterial enumeration

Mice were sacrificed at predetermined intervals (see diagram in **Figure 1** A). Lungs were aseptically removed, weighed and placed in 2.5 ml sterile PBS for 24-48 h at 4°C, examined for gross pathology and manually homogenized. Homogenates were serial-diluted, and 0.5 ml plated on Middlebrook 7H11 agar (Difco, Thermo Fisher Scientific, Waltham, MA, USA) supplemented with 10% (v/v) OADC, 0.5% (v/v) glycerol, 10 mg/ml cycloheximide, 50 mg/ml carbenicillin, 25 mg/ml polymyxin B and 20 mg/ml trimethoprim (Sigma-Aldrich, St. Louis, MO, USA). Plates were incubated at 37°C for 3-4 weeks before colonies were counted. For relapse studies, plates were checked after 4 and 8 weeks to allow for proliferation of slow-growing bacilli. Colony numbers were adjusted by the plating and dilution factors to estimate the total colony-forming units (CFUs) and are reported as absolute or log-transformed CFUs per lung.

Histopathology

For histology, intact lungs were fixed by immersion in 10% neutral-buffered formalin for 48 hours, paraffin-embedded, sectioned and stained with hematoxylin and eosin (H&E) stain. Slides were digitally scanned at 40x on an Aperio AT turbo scanner console version 102.0.7.5 (Leica Biosystems, Vista, CA, USA). Image files were transferred using Concentriq for Research version 2.2.4 (Proscia Inc, Philadelphia, PA, USA) and visualized using Aperio ImageScope version 12.4.0.5043 (Leica Biosystems Pathology Imaging, Buffalo Grove, IL, USA).

^{18}F-FDG PET/CT Imaging

For ^{18}F-FDG PET/CT, six animals from the RHZ or RHZ + ISRIB groups were sequentially imaged at the start of treatment and after 2, 4 and 6 months of treatment, inside in-house developed, sealed biocontainment devices compliant with BSL-3 isolation (25, 28, 35). Mice were fasted for 8 h prior to imaging. Each animal was injected 6.9 ± 0.69 MBq of ^{18}F-FDG intravenously via the tail vein. A 15-minute PET acquisition and subsequent CT were performed using the nanoScan PET/CT (Mediso, Arlington, VA, USA). For each animal, six regions of interest (ROIs) were manually selected using CT as a guide and applied to the PET dataset using VivoQuant™ 2020 (Invicro, Boston, MA, USA) for visualization and quantification. Mean lung ^{18}F-FDG PET activity was calculated for each mouse (n=6 per treatment group) as the average activity of all VOIs normalized by injected dose (% ID/cc). Data was analyzed non-parametrically and is presented on a linear scale as median and interquartile range (IQ).

Statistical analysis

Statistical analyses were performed using Prism version 9.2.0 for Windows (GraphPad, San Diego, CA, USA). Statistical tests used are indicated in the figure legends. CFU counts were $\log_{10}$-transformed prior to analysis up until 4 months of treatment, after which absolute counts were compared. Differences in bacterial burden between RHZ and RHZ + ISRIB groups were assessed by unpaired, two-tailed t-test. Relapse proportions were compared by two-sided Fisher's exact test and are expressed as the fraction of relapsed over the total number of mice per group. 95% confidence intervals for relapse proportions were computed by hybrid Wilson/Brown method. Differences in 18F-FDG PET activity were evaluated by nonparametric Mann-Whitney test. A p value below 0.05 was considered significant. Data represent mean ± SEM unless otherwise

indicated. Histology images were assembled using the open-source vector graphics editor Inkscape for Windows v. 0.92.4 (Boston, MA, USA).
